# Visualization of anatomical structures in the fetlock region of the horse using cone beam computed tomography in comparison with conventional multidetector computed tomography

**DOI:** 10.3389/fvets.2023.1278148

**Published:** 2024-01-05

**Authors:** Jonathan Bierau, Antonio M. Cruz, Christoph Koch, Gabriel Manso-Diaz, Kathrin Büttner, Carsten Staszyk, Michael Röcken

**Affiliations:** ^1^Equine Clinic (Surgery, Orthopedics), Justus-Liebig-University Giessen, Giessen, Germany; ^2^Department of Clinical Veterinary Medicine, Vetsuisse Faculty, Swiss Institute of Equine Medicine (ISME), University of Bern, Bern, Switzerland; ^3^Faculty of Veterinary Medicine, Department of Animal Medicine and Surgery, Universidad Complutense de Madrid, Madrid, Spain; ^4^Unit for Biomathematics and Data Processing, Justus-Liebig-University Giessen, Giessen, Germany; ^5^Institute of Veterinary-Anatomy, -Histology, and -Embryology, Faculty of Veterinary Medicine, Justus-Liebig-University Giessen, Giessen, Germany

**Keywords:** horse, fetlock, cone beam computed tomography, anatomy, diagnostic

## Abstract

**Introduction:**

Cone-beam computed tomography (CBCT) is regarded as a convenient and suitable alternative to conventional computed tomography. However, in the horse, the quality of obtained data sets needs to be evaluated. Therefore, the aim of this study was to compare the visibility and accessibility of clinically relevant anatomical structures displayed in CBCT and conventional multidetector computed tomography (MDCT).

**Materials and methods:**

Twenty-nine limbs from horses euthanized for reasons unrelated to this study were used. Native and intraarticular contrast scans of the fetlock (CBCT vs. MDCT) were performed. The visibility and accessibility of selected anatomical structures were blindly scored by three independent experienced observers using a scoring system previously reported and adapted to the fetlock joint.

**Results:**

Only minor differences between CBCT and MDCT were identified concerning the diagnostic quality of images for osseous structures. Soft tissue structures were better evaluated on MDCT images. In CBCT as well as in MDCT articular cartilage could only be visualized after intraarticular injection of contrast medium.

**Discussion/conclusion:**

Cone beam computed tomography of the fetlock is a useful and reliable diagnostic tool when evaluating osseous structures and delineating articular cartilage with contrast medium. However, this modality is limited for assessing soft tissues structures.

## 1 Introduction

Pathologies of the equine fetlock joint are some of the most common orthopedic reasons for veterinary consultation (1, 2). Thirty-three percent of thoroughbreds between 2 and 3 years of age suffer from fetlock joint disease before the end of their second or even first racing season (3). Radiography is commonly used for determining the location, the type of lesion, and the severity of the pathological changes. However, the complex anatomy of the fetlock joint and the inherent lack of spatial resolution of conventional radiographic imaging that entails superimposition of relevant anatomical structures complicates or even hampers an accurate assessment of pathological changes (4). To overcome these limitations, computed tomography (CT) has become an important diagnostic tool to assess the equine fetlock joint and adjacent structures (5). Two different CT technologies are currently available for imaging of the equine fetlock joint: conventional fan-beam multidetector computed tomography (MDCT), and cone-beam computed tomography (CBCT) (6). In MDCT, images are acquired using a narrow, fan-shaped X-ray beam. The area of interest is imaged slice by slice along the patient’s *z*-axis (7). In contrast, CBCT uses a cone-shaped X-ray beam and a large flat panel detector. Volumetric data are obtained with multiple projections from a single rotation around the region of interest. One advantage of all flat-panel detector-based CBCT imaging is the smaller pixel size of most detectors compared to conventional CT, which currently ranges from 120 to 150 μm, and a higher spatial resolution of 1.5 line pairs (LP)/mm to 3 Lp/mm, which is superior compared to a conventional CT with 1.2–1.4 Lp/mm (8). The known limitations of CBCT in comparison with MDCT are the lower contrast resolution, due to increased scattering and the absence of detector conduction septa, as well as the inherent increased susceptibility of CBCT to motion- and cone beam-related artifacts (9). Furthermore, soft tissue contrast has been reported to be poorer with CBCT (10), and therefore, MDCT is considered superior for soft tissue evaluation. Compared with stationary MDCT, mobile CBCTs, such as the O-arm® (Medtronic), have several advantageous features for clinical application in equine referral centers. The highly mobile CBCT scanners neither require a fixed installation nor a separate, specific power supply. The gantry of the O-arm® is readily adjusted in all three directions and can be tilted around its horizontal and vertical axis. Therefore, this mobile CBCT is easily moved from one room to another (such as operating room, diagnostic room), and its gantry can be positioned around the anatomic region of interest, often avoiding the need for general anesthesia. Moreover, the acquisition costs for a mobile CBCT are normally lower compared to those for most MDCTs. Therefore, a less expensive CT examination can be offered to clients. Nonetheless, considering the differences in image quality, in particular, the poor soft-tissue resolution of CBCT imaging, additional comparisons of MDCT and different makes of CBCT scanners in equine practice are needed.

This study compared the output of diagnostically useful imaging data of a mobile CBCT scanner with that of an MDCT scanner for the assessment of clinically relevant anatomical structures of equine fetlock.

## 2 Materials and methods

### 2.1 Cadaveric specimens

Twenty-nine limbs from nine horses euthanized for reasons unrelated to this study at the Department of Equine Surgery and Internal Medicine at the Clinic for Horses of the Justus-Liebig-University in Giessen were used (Table 1). Seven limbs of nine horses were not suitable to be used in this study due to laceration and other lesions received *post mortem*. Within 1 h after euthanasia, the limbs were sectioned at the level of the carpus or tarsus. In the following, only the anatomical terms for the directions and structures of the forelimb (e.g., palmar, metacarpus) are used. Hindlimbs were assessed accordingly. Subsequently, the metacarpo−/tarso-phalangeal joints were radiographed (four projections were obtained: dorso-palmar, latero-medial, dorsolateral-palmaromedial oblique, and dorsomedial-palmarolateral oblique views) using a high-frequency generator (Siemens Optitop 150/40/80, 68 kVp and 2.0 mAs) and a DR flat panel detector (Fujifilm, FDR D-EVO II C24). Subsequently, the limbs were stored at 4°C before MDCT and CBCT examinations, which were performed within 24 h after euthanasia.

**Table 1:** Patient data.

Horse number	Fetlock number	Age (years)	Breed	Sex	Weight (kg)	Leg	Reason for euthanation
1	1	7	Thoroughbred	Mare	386	Left forelimb	Colic
1	2	7	Thoroughbred	Mare	386	Right forelimb	Colic
1	3	7	Thoroughbred	Mare	386	Left hindlimb	Colic
1	4	7	Thoroughbred	Mare	386	Right hindlimb	Colic
2	5	7	Haflinger	Mare	500	Left Forelimb	Hoof abscess
2	6	7	Haflinger	Mare	500	Right forelimb	Hoof abscess
2	7	7	Haflinger	Mare	500	Left hindlimb	Hoof abscess
2	8	7	Haflinger	Mare	500	Right hindlimb	Hoof abscess
3	9	10	Warmblood	Mare	535	Left forelimb	Colic
3	10	10	Warmblood	Mare	535	Right forelimb	Colic
4	11	12	Warmblood	Gelding	600	Left forelimb	Ataxia
4	12	12	Warmblood	Gelding	600	Right forelimb	Ataxia
4	13	12	Warmblood	Gelding	600	Left hind limb	Ataxia
4	14	12	Warmblood	Gelding	600	Right hindlimb	Ataxia
5	15	15	Warmblood	Mare	544	Left forelimb	Colic
5	16	15	Warmblood	Mare	544	Right forelimb	Colic
5	17	15	Warmblood	Mare	544	Left hindlimb	Colic
5	18	15	Warmblood	Mare	544	Right hindlimb	Colic
6	19	15	Warmblood	Gelding	630	Left forelimb	Colic
6	20	15	Warmblood	Gelding	630	Right forelimb	Colic
6	21	15	Warmblood	Gelding	630	Left hindlimb	Colic
6	22	15	Warmblood	Gelding	630	Right hindlimb	Colic
7	23	20	Fjord horse	Mare	450	Left hindlimb	Colic
7	24	20	Fjord horse	Mare	450	Right hindlimb	Colic
7	25	23	Pony	Gelding	528	Left forelimb	Colic
7	26	23	Pony	Gelding	528	Right forelimb	Colic
8	27	23	Pony	Gelding	528	Left hindlimb	Colic
8	28	23	Pony	Gelding	528	Right hindlimb	Colic
9	29	25	Half-breed	Mare	550	Right forelimb	Colic

### 2.2 CBCT and MDCT scans

The CBCT scanner used in the present study was designed and FDA-approved for use in a surgical environment (O-arm®, Medtronic Inc.). The MDCT scans were performed using a helical 16-slice MDCT scanner (Somatom® Definition AS Siemens, Erlangen, Germany). For the CBCT investigation, 120 kV, 64 mAs, and field of view (FOV) 20 cm were used. The MDCT acquisition parameters were 130 kV, 173 mAs, 0.75 mm slices, FOV of 25.5 cm, soft tissue and bone algorithm.

Each metacarpo-/tarso-phalangeal joint was scanned with both modalities, the CBCT and the MDCT. For all scans, limbs were positioned in dorsal recumbency. First, a native scan was generated with each modality. Subsequently, arthrograms were performed for each metacarpo-/tarso-phalangeal joint and the imaging procedure was repeated. For the arthrograms, each joint was injected through a dorsal approach and with a 20-G needle (Stercan®, Braun). A 1:1 mixture of contrast medium containing iobitridol (Xenetix® 300, Guerbet, Sulzbach, Germany) and isotonic saline solution 0.9% (Braun Ecofl from B. Braun Melsungen AG) was injected until the joint was ballooned (volume 20 mL). To achieve an even distribution of the injected contrast solution, each joint was flexed 20 times.

### 2.3 Image evaluation

**Figure 1:**
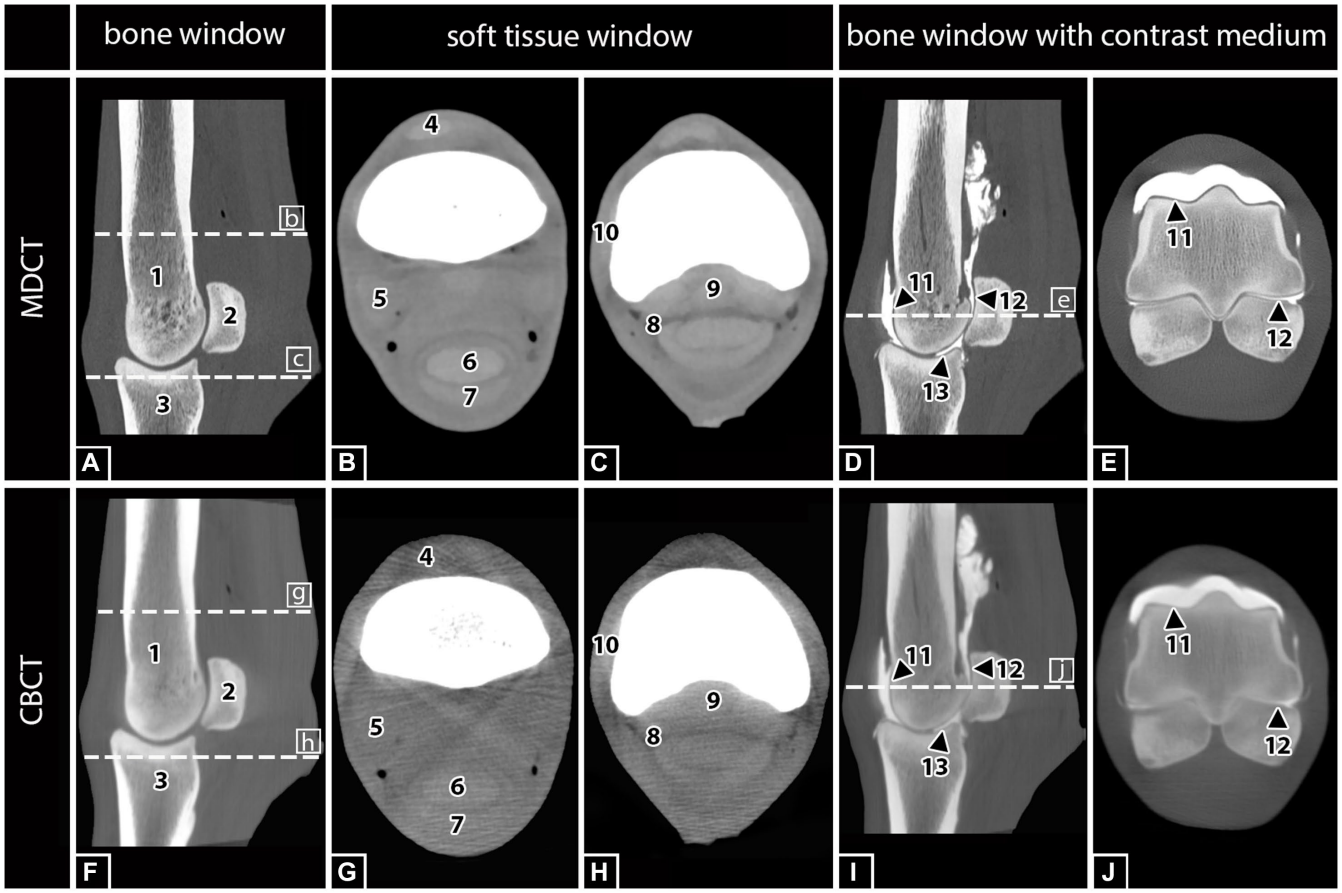
MDCT and CBCT images of the right hind fetlock region of a 15-year-old gelding. The visibility and assessability of the listed anatomical structures were tested in MDCT and CBCT images with and without contrast medium injection: (1) Third metatarsal bone (MTIII), (2) proximal sesamoid bones (PSB), (3) proximal phalanx (PP), (4) common digital extensor tendon (CDET), (5) suspensory ligament (SL), (6) deep digital flexor tendon (DDFT), (7) superficial digital flexor tendon (SDFT), (8) digital flexor tendon sheath (DFTS), (9) sesamoidean ligaments (SAL), (10) collateral ligament of the fetlock (CL), (11) cartilage of MTIII, and (12) cartilage of PSB, and (13) cartilage of PP.

Cone beam computed tomography and MDCT scans were rendered using a DICOM viewing software (DICOM Horos® viewer). The different slice planes were created using a multiplanar reconstruction (MPR) tool. Each limb was randomly assigned a case number. Assignment of the different modalities (CBCT or MDCT) to the respective limb was possible for the observers. The CBCT images were evaluated first, followed by the MDCT images. All observers were experienced in assessment of CBCT and MDCT images. The scoring system was explained in a meeting before the evaluation started. Per observer, each limb was evaluated once. The images were evaluated by two board-certified equine surgeons (CK, AC) and one board-certified large animal radiologist (GMD). The following anatomical structures (Figure 1) were evaluated using a modified scoring system according to Vallance et al. (11).

Osseus structures:

- Third metacarpal/−tarsal bone (MCIII/MTIII)
- Proximal sesamoid bones (PSB)
- Proximal phalanx (PP)

Soft tissue structures:

- Common digital extensor tendon (CDET)
- Suspensory ligament (SL)
- Deep digital flexor tendon (DDFT)
- Superficial digital flexor tendon (SDFT)
- Digital flexor tendon sheath (DFTS)
- Sesamoidean ligaments (SAL)
- Collateral ligament of the fetlock (CL)

Articular structures:

- Cartilage of MCIII/MTIII
- Cartilage of PSB
- Cartilage of PP

**Figure 2:**
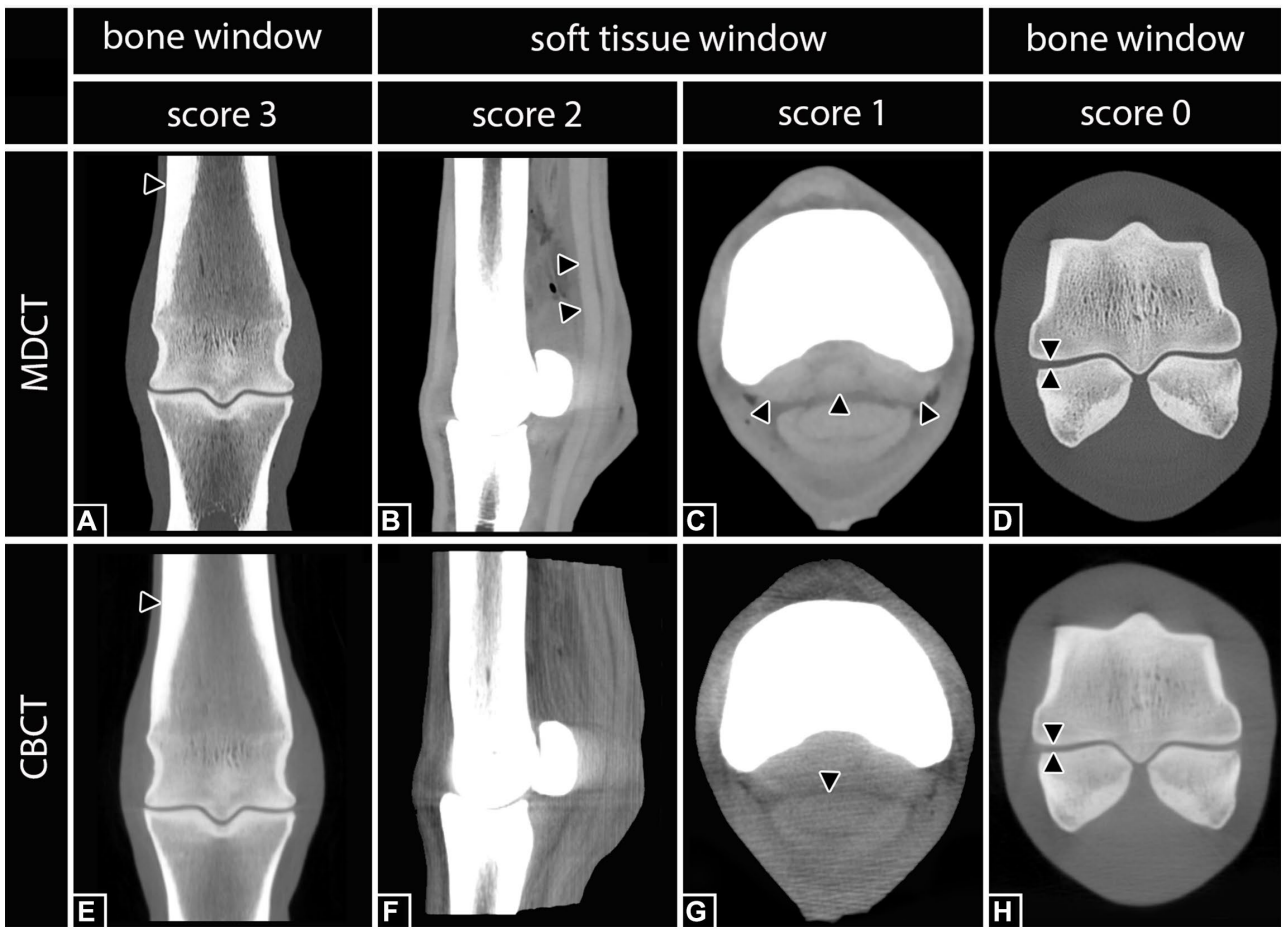
Definition of the applied scoring system according to Vallance et al. (11) including examples of differently scored anatomical structures. Right hind fetlock region. Score 3: **(A,E)** The third metatarsal bone (black arrows) was clearly visualized and delineated by location, shape, attenuation, size, and margin in both, MDCT and CBCT. Score 2: **(B)** In MDCT, the deep digital flexor tendon (black arrows) was clearly identified by its location, shape, and attenuation, but the margins were not clearly delineated. In the corresponding CBCT image **(F)**, none of the structures was scored 2. Score 1: **(C)** In MDCT, the sesamoidean ligaments (black arrows) were poorly visualized, but detectable, and were identified by location and attenuation but not by margins, shape, or size. In the corresponding CBCT image **(G)**, the deep digital flexor tendon (black arrow) was scored 1. Score 0: **(D,H)** Although the lining of the cortical bone of the third metatarsal bone and the proximal sesamoid bones is clearly visible, the belonging cartilage was not visible.

Each aforementioned structure was assigned a visual assessment score of 0–3 using subjective criteria for visibility. A score of 0 indicated that the structure was not visible. A score of 1 indicated that the structure was poorly visualized, but detectable, and was identified by its location and attenuation but not by margins, shape, or size. A score of 2 represented a structure that was clearly identified by its location, shape, and attenuation, but the margins were not clearly delineated. A score of 3 indicated that the anatomic structure was well visualized and clearly delineated by location, shape, attenuation, size, and margins (11) (Figure 2).

### 2.4 Statistics

A comparison between MDCT native, CBCT native, MDCT contrast, and CBCT contrast for the anatomical visualization of osseus structures, soft tissue structures, and articular structures was performed. A descriptive evaluation was performed (Table 2). Spearman’s rank correlation was used to determine the agreement of CBCT with MDCT. An R_s_ value of >0.4 represents acceptable agreement, and R_s_ values >0.7 represent good agreement. Inter-observer agreement was determined by the percent agreement. The closer the value is to 1, the more exact is the match. The lower limit of the 95% confidence interval of the mean value and the upper limit of the 95% confidence interval of the mean value are indicated in brackets.

**Table 2:** Anatomical visualization score and technique comparison of cone beam computed tomography (CBCT) and conventional multidetector computed tomography (MDCT).

		Cartilage			Soft tissue					Ligaments	
Technique	Statistic	Cartilage MCIII/MTIII	Cartilage PSB	Cartilage PP	CDET	SL	DDFT	SDFT	DFTS	SAL	CL
MDCT nativ	Median (range)	0.00 (0–0)	0.00 (0–0)	0.00 (0–0)	2.00 (0–3)	2.00 (0–3)	2.00 (1–3)	1.00 (0–2)	0.00 (0–3)	1.00 (0–3)	0.00 (0–2)
*n* = 87											
MDCT contrast	Median (range)	3.00 (0–3)	3.00 (2–3)	3.00 (0–3)	2.00 (0–3)	2.00 (1–3)	2.00 (1–3)	2.00 (1–3)	1.00 (0–3)	1.00 (0–3)	0.00 (0–2)
*n* = 87											
CBCT nativ	Median (range)	0.00 (0–0)	0.00 (0–0)	0.00 (0–0)	0.00 (0–2)	1.00 (0–2)	1.00 (0–2)	1.00 (0–2)	0.00 (0–2)	0.00 (0–2)	0.00 (0–1)
*n* = 87											
CBCT contrast	Median (range)	3.00 (0–3)	3.00 (2–3)	3.00 (0–3)	0.00 (0–1)	1.00 (0–2)	1.00 (0–2)	1.00 (0–2)	0.00 (0–1)	0.00 (0–2)	0.00 (0–1)
*n* = 87											
Comparisons											
MDCT nativ vs.	Spearman-	*r* = .*	*r* = .*	*r* = .*	*r* = −0.07	*r* = 0.17	*r* = 0.20	*r* = 0.21	*r* = 0.68	*r* = 0.69	*r* = 0.14
CBCT nativ	rangkorrelation										
	p-Wert	*p* = .^*^	*p* = .^*^	*p* = .^*^	*p* = 0.52	*p* = 0.12	*p* = 0.06	*p* = 0.05	*p* ≤ 0.01	*p* ≤ 0.01	*p* = 0.19
MDCT contrast vs.	Spearman-	*r* = 0.85	*r* = −0.02	*r* = 0.85	*r* = −0.07	*r* = −0.02	*r* = −0.03	*r* = −0.13	*r* = 0.68	*r* = 0.66	*r* = 0.14
CBCT contrast	rangkorrelation										
	*p*-Wert	*p* ≤ 0.01	*p* = 0.83	*p* ≤ 0.01	*p* = 0.53	*p* = 0.86	*p* = 0.81	*p* = 0.22	*p* ≤ 0.01	*p* ≤ 0.01	*p* = 0.20

^*^The correlation coefficient could not be calculated, because there was not enough variance in the present data. MCIII/MTIII, Third metacarpal/tarsal bone; PSB, Proximal sesamoid bones; PP, Proximal phalanx; CDET, Common digital extensor tendon; SL, Suspensory ligament; DDFT, Deep digital flexor tendon; SDFT, Superficial digital flexor tendon; DFTS, Digital flexor tendon sheath; SAL, Sesamoidean ligaments; CL, Collateral ligament of the fetlock.

## 3 Results

### 3.1 Comparison of native MDCT and CBCT images

For all assessed bony structures (MCIII/MTIII, PSB, PP; *n* = 87), no significant differences between CBCT and MDCT were detected in terms of the described scoring criteria. All bony structures scored 3, with an interobserver agreement of 100%; Spearman correlation *r* = 1. The same high interobserver agreement of 100% and correlation of MDCT to CBCT (*r* = 1) could be obtained for the cartilage. It was not identified, neither in MDCT nor CBCT native scans without contrast medium (score = 0). A weak to moderate correlation of soft tissue structures (CDET, SL, DDFT, SDFT, and FTS) between MDCT and CBCT was noted (*n* = 145; *r* = 0.30–0.55; Table 2). The MDCT always obtained a better representation of soft tissue structures (CBCT mean = 0.58 vs. MDCT mean = 1.72). Interobserver agreement was 71% on the mean for soft tissue structures for MDCT, except for FTS with only 24%. The CBCT had a mean agreement of 55%. Ligaments (CL, SAL; *n* = 58) were poorly detected in both modalities (score = 0–1; *r* = 0.37–1.0). The observer agreement for ligaments was highly variable. The CL showed an agreement of 40% on MDCT and 98% on CBCT. The SL showed an agreement of 15% on MDCT and 44% on CBCT.

### 3.2 Comparison of MDCT native with MDCT contrast/CBCT native with CBCT contrast

Bony structures (MCIII/MTIII, PSB, PP; *n* = 87) were readily identified, without or with contrast medium (score = 3; *r* = 1). However, all observers reported a subjective decrease in bony visualization after contrast injection on MDCT. A significant improvement of the visualization of cartilage was shown for the contrast agent in MDCT and CBCT (score = 2–3; *r* = 0.9). The presence of contrast agent had no significant impact on the visualization of the soft tissue structures with neither modality (different after arthrography: MDCT score + 0.01 to −0.14; CBCT score + 0.03 to −0.17).

## 4 Discussion

The present study was performed to evaluate the visualization of anatomical structures of the equine fetlock region by CBCT and MDCT, using a scoring system. Only minor differences between CBCT and MDCT were obtained concerning the visualization of osseous structures. The MDCT images showed superior quality regarding the assessment of soft tissue structures. In CBCT as well as in MDCT, articular cartilage thickness was well visualized after the injection of contrast medium.

### 4.1 HU usability for the assessment of anatomical structures

A strong correlation between gray levels of CBCT images and Hounsfield units (HU) defined in MDCT images has already been described (12). However, the strong interference of artifacts in CBCT does not allow a complete transfer of CBCT gray levels in HU. The limited use of HU with CBCT imaging complicates the identification of structural changes in CBCT images. However, CBCT imaging allows for a detail-rich visualization of high-contrast structures, such as bones, teeth, and air-filled cavities. Traditionally, bone quality parameters and classifications were primarily based on bone density and can readily be estimated using HU derived from MDCT datasets. Attempts to calibrate HU in CBCT leads to inaccurate HUs in some systems even when using a phantom with known bone density, mainly because the reconstruction algorithm is inconsistent due to the system or becomes inconsistent due to artifacts (13). All in all, important technical aspects differing between CBCT and MDCT need to be considered, including the restrictions in field/volume of view and the increased scatter radiation in CBCT imaging. Furthermore, differences and limitations of the currently used reconstruction algorithms between MDCT and CBCT complicate direct image quality comparisons and the use of quantitative gray values.

### 4.2 Reasons for poorer soft tissue visualization

The results of the present study confirm that soft tissue visualization is poorer with CBCT compared to MDCT. However, there are software packages and other technical solutions that allow to improve soft tissue visualization in CBCT imaging. In studies using human cadavers, a special soft tissue filter (Hann filter) was used in CBCT imaging, which significantly improved the visualization of soft tissue structures (14). Nevertheless, even with the filter, soft tissue visualization in CBCT did not reach the quality of MDCT. In order to overcome the deficient representation of soft tissue structures in CBCT and MDCT an additional ultrasonography examination is recommended (15). Another problem inherent to CBCT imaging and leading to poorer soft tissue particularly in the fetlock joint region, with a high ratio of dense cortical bone to soft tissue structures, is scatter radiation from the CBCT x-ray source. Scatter radiation increases the image noise and decreases the contrast-to-noise ratio, thus contributing to a poor soft tissue resolution (16). The MDCT technique is capable of producing more homogeneous images with higher contrast levels compared to CBCT (17).

### 4.3 Limited FOV

The limited volume of view (19,806 cm^3^) of the CBCT does not appear to be a disadvantage in the fetlock region. However, for CBCT examinations in more voluminous regions, such as the stifle joint, the limited FOV may require the acquisition and diagnostic screening of multiple scans to allow for the visualization of the entire region of interest. Again, the use of adequate software that automatically merges and assembles separate scans will, at least partially, overcome this limitation of CBCT imaging and make it more efficient to read. Alternatively or in addition, blending the primary beam to the minimum required to display the ROI would be a means of reducing scatter radiation and artifacts and thereby improving image quality (18). However, this is not an option with most CBCT scanners, which have no or only limited adjustability of the FOV.

### 4.4 Influence of arthrography

The use of contrast-enhanced CT imaging has been widely evaluated in horses for vascular (19, 20) as well as synovial (21) structures. Contrast CT imaging has been identified as an effective procedure to enhance several anatomic structures and related pathologies (such as cartilage defect and synovial hernia). In both modalities used for this investigation, CBCT and MDCT, contrast medium enhanced the visualization of the articular surface in the fetlock joint. However, only a minor improvement was recorded for the visualization of soft tissue structures. For example, low-grade, poor visualization of the SL, DDFT, and SDFT was noted in both CBCT and MDCT imaging when combined with contrast filling of the metacarpo-/metatarso-phalangeal joint (Table 2). This may have worsened the soft tissue delineation at least partially, contributing to streak artifacts caused by the contrast agent (22). However, and regardless of the modality, the cartilage visualization was only possible after the articular injection of contrast medium. While contrast medium diffused and accumulated in most areas of the metacarpo-/metatarso-phalangeal joint, it did not accumulate in the area of the sagittal ridge and sagittal groove. A similar insufficient visualization in these regions has previously been reported for MDCT contrast imaging (23). In the present cadaver study, the homogenous distribution of contrast medium in all aspects of the metacarpo-/metatarso-phalangeal joint was hampered despite the limbs being non-weight-bearing for the contrast study. Studies investigating the effects of weight-bearing, the degree of joint movement, extension or flexion, and/or different volumes and dilutions of contrast medium are therefore needed to optimize the visualization of cartilage and cartilage lesions using CT imaging.

### 4.5 Limitations of subjective evaluations

A scoring system in combination with an interobserver agreement analysis was used to determine the effect of the observer on the visualization of anatomical structures in CBCT images compared to MDCT images. Superior visualization quality (score = 3) and highest interobserver agreement were recorded for bony structures (with and without contrast medium) in CBCT as well as in MDCT images.

Inter- and intraobserver agreement was excellent for the identification of structures that were clearly not visible (score 0) or indicated that the anatomic structure was well visualized and clearly delineated by location, shape, attenuation, size, and margins (score 3) for MDCT (native/contrast) and CBCT (native/contrast). Examples include bony structures or cartilage post contrast. The interobserver agreement for soft tissue structures was reduced, irrespective of the modality used. This can be explained by the short range of possible scores (1–2) for soft tissue structures. In the absence or presence of clearly identifiable structures, the scoring system will result in a more moderate interobserver and intraobserver agreement. This can lead to an increased difficulty in evaluating the affected structures, resulting in a higher observer variation, as already observed in human medicine (24).

### 4.6 Outcomes of existing studies of comparisons CBCT vs. MDCT

Recently, comparative studies between MDCT and CBCT imaging have been performed to compare the diagnosis of pathological changes in the equine head, cervical area, and the fetlock (25–28). In conclusion, the CBCT results were similar to those obtained via conventional MDCT for the majority of dental abnormalities; however, pulp abnormalities were not reliably identified using CBCT, potentially limiting its clinical use for detecting endodontic disease in its current form (26).

A study that performed a validation of standing CBCT for the diagnosis of subchondral fetlock pathology in the thoroughbred racehorse showed that intermodality correlation (MDCT vs. CBCT) and concordance were significantly consistent for all variables interpreted by the radiologist. Intermodality correlation was significantly consistent for 19 out of 25 variables after interpretation by the surgeon. The investigators concluded that standing CBCT is a valid diagnostic modality for identifying subchondral bone lesions in metacarpo-/metatarso-phalangeal joints in horses (27). This is in agreement with our results, which indicate that CBCT imaging provides images of diagnostic value, comparable to MDCT imaging, for bony structures in the area of the fetlock. In another study, two types of heterotopic mineralization (ossification, including abaxial avulsion fracture of the proximal sesamoid bones, and ligament and tendon mineralization) were reliably detected using CBCT imaging of the equine fetlock region (25). Likewise, the general suitability of CBCT imaging for the diagnostic assessment of bony structures and bone-related pathologies has been shown by numerous comparative studies on human skeletal structures (29, 30). Using clinically applied protocols, both CBCT and MDCT imaging produced diagnostic images for the assessment of the human pelvis, head, and neck. The images were assessed for various quality parameters to determine which modality produced the most expressive images to detect irregularities of bony and soft tissue structures. The images produced by MDCT were of superior quality in clarity, uniformity, anatomical accuracy, low contrast resolution, and delivery of a lower dose to the patient (9).

Although the image quality and diagnostic value of the chosen imaging modality are of highest importance for the selection of an imaging technique, technical features, and profitability cannot be disregarded. Both MDCT and CBCT can produce scans of the head, cervical spine, and entire appendicular skeleton. However, CBCT has the advantage of being a mobile and diagnostic imaging modality for orthopedic examinations of the standing limb in horses and can be performed in a timely manner with limited acquisition attempts at low cost (6, 31).

### 4.7 Usability and setting options CBCT

Despite the limitations of the technology, CBCT consistently provided clinically relevant new findings in a variety of cases, either as a new diagnosis or as supportive new findings for a range of cartilage, bone, and soft tissue pathologies. The horse is sedated for the examination and placed in a stock. A carbon table is placed in front of the stand. The limb is positioned either forward in the case of the forelimb or backward in the case of a hind limb using loops. On the standing sedated horse an examination up to carpus and tarsus is possible (32). In general anesthesia up to elbow and hip in small horses. Movement artifacts lead to repetition of the scans. This can occur more frequently in painful or uncooperative patients who are recommended to be examined under general anesthesia. A CBCT of the skull can also be performed in a standing position. A sedated horse, with earplugs and lying on a vacuum cushion on a carbon table, can be examined without the need for restraining staff (33). Likewise, CBCT can be useful as a preoperative planning tool. The CBCT applied in this study has three options available for making a scan. We chose High-Definition 3D (HD3D), which offers a better image quality than the standard 3D or low-dose display (34). As a result, approximately 745 projections were recorded. This allows for a higher spatial and contrast resolution and increases the signal-to-noise ratio, resulting in a better resolution. Exposition to radiation also plays an important role for the person performing the examination as she/he needs to be present in the examination room for particular indications on standing sedated horse. A low radiation exposure for CBCT has been described due to the smaller FOV and lower dose (35). This is true for an examination under general anesthesia. However, this advantage is significantly truncated in whole examination in standing sedated horses because usually, several scans are needed due to clustered motion artifacts (33). It is fair to assume that the latter may, in some cases, lead to increased radiation exposure. The CBCT has a rotation time in the HD module of 27 s, in contrast to the MDCT used for this study, which requires only approximately 1 s. With the MDCT setting we used, total scan time was 27 s. Considering the scan time of both systems, CBCT has a relatively long rotation time, in which a single movement leads to artifacts in the entire scan. In contrast, motion only affects the area immediately scanned on the MDCT.

### 4.8 Limitation

Study was conducted on cadaveric limbs. In order to minimize artifacts due to post mortal changes, limbs were scanned as soon as possible after euthanasia (max. 24 h). The scan of the dissected limbs resembles a scan under general anesthesia, rather than a CBCT scan in a standing sedated horse. Therefore, the problem of motion artifacts as described elsewhere (33) was not assessed in this study.

### 4.9 Conclusion

The diagnostic value of CBCT imaging of clinically relevant bony structures in the fetlock region is equivalent to that of images obtained by MDCT. Concerning technical features, practicability, and operating costs, CBCT is regarded as a cost-effective and mobile alternative to MDCT imaging, providing cross-sectional and 3D image data of the fetlock region to detect pathological changes in bony structures and cartilage after contrast injection. However, the visualization of soft tissue structures in the fetlock region by CBCT remains inferior compared to that obtained via MDCT.

## Data availability statement

The original contributions presented in the study are included in the article/supplementary material, further inquiries can be directed to the corresponding author.

## Ethics statement

Ethical review and approval was not required for the animal study because according to German legislation, the post mortal collection of specimens does not need any permission of the animal welfare authority. Written informed consent was obtained from the owners for the participation of their animals in this study. The studies were conducted in accordance with the local legislation and institutional requirements. Written informed consent was obtained from the owners for the participation of their animals in this study.

## Author contributions

JB: Writing – original draft. AC: Investigation, Writing – review & editing. CK: Investigation, Writing – review & editing. GM-D: Investigation, Writing – review & editing. KB: Data curation, Writing – review & editing. CS: Supervision, Writing – review & editing. MR: Supervision, Writing – review & editing.
